# Five‐Year Follow‐Up of Patients With Histologically Aggressive Basal Cell Carcinoma Treated With Interferon

**DOI:** 10.1155/jskc/8852284

**Published:** 2026-06-29

**Authors:** Vladimir Sanchez Linares, Vicente Eloy Fardales Macías, Iraldo Bello Rivero

**Affiliations:** ^1^ University of Medical Sciences of Sancti Spíritus, Circunvalación s/n, Sancti Spíritus, Cuba; ^2^ Center for Genetic Engineering and Biotechnology, Havana, Cuba, cigb.edu.cu

**Keywords:** aggressive histology, basal cell carcinoma, interferons, tumor recurrence

## Abstract

**Background:**

Basal cell carcinomas (BCCs) with aggressive histology are infiltrative, destructive, aggressive, and metastatic tumors. Aggressive variants include morpheaform, infiltrative, metatypical or basosquamous, micronodular, and mixed subtypes. Selecting the appropriate treatment is crucial to eliminate this type of tumor and avoid complications such as mutilation and deformities when located in the facial region, as well as tumor recurrence. Interferons may represent a therapeutic option because of their curative potential and long‐term cosmetic effects.

**Material and Methods:**

A descriptive, longitudinal, observational case‐series study was conducted, including patients with a histopathological diagnosis of aggressive BCC. Perilesional HeberFERON was administered for three weeks, and patients were then followed for 5 years. The main outcome variables were cure at 16 weeks and recurrence at 5 years.

**Results:**

Twenty‐three patients were included, and one discontinued treatment. After therapy, 14 (63.6%) patients achieved cure (complete response [CR]). Those who did not achieve a CR underwent surgery and were declared cured. All 22 patients were followed for five years; two (9.0%) were lost to follow‐up. Survival analysis showed that 86.4% (95% CI, 74%–99%) had the probability of remaining recurrence‐free at five years.

**Conclusions:**

HeberFERON can be considered a first‐line treatment for BCC with aggressive histology and as a therapy prior to subsequent surgery.

## 1. Introduction

Basal cell carcinoma (BCC) is the most common nonmelanoma skin cancer and accounts for 80% of malignant skin lesions in adults older than 50 years with fair skin phototypes. It is located mainly in sun‐exposed areas. It originates from epidermal cells of the hair follicles or from the basal cells of the immature pluripotent epidermis. The main risk factor for the development of BCC is chronic exposure to ultraviolet radiation [1–3].

According to histology, BCC may be classified as aggressive or nonaggressive. Aggressive histological variants include morpheaform, infiltrative, met atypical or basosquamous, micronodular, and mixed. Morpheaform or sclerosing BCC is characterized by prominent stromal fibrosis; the nests of basaloid cells are thin and do not show peripheral palisading or retraction clefts with the stroma, it affects the deep dermis, and perineural infiltration is not uncommon. Infiltrative BCC is similar to morpheaform BCC but is not associated with a fibrotic stroma [4–6].

Micronodular BCC is composed of small, poorly cohesive rounded basaloid epithelial islands without peripheral palisading or retraction clefts between the epithelium and the stroma, with a histological infiltrative capacity greater than what is suggested clinically; it is frequently located in the perinasal area. Basosquamous carcinoma is composed of areas of basaloid cells with infiltrative growth and areas of squamous cells with an intermediate transition zone representing differentiation from one form to the other. It is a tumor with a high tendency toward recurrence and metastasis. Mixed BCC is diagnosed when different patterns converge in the same tumor [4–7].

Histologically aggressive BCCs are locally infiltrative, destructive, aggressive, and metastatic and are sometimes underestimated by dermatologists [4, 7]. Hence, the importance of therapy aimed at eliminating the tumor completely and preventing recurrences or the previously mentioned complications. Although surgery is the treatment of choice for excising this tumor, the fact that it is considered a high‐risk BCC breaks the golden rule of therapy, and sometimes, given its characteristics, it is impossible to resect it completely [7].

Immunotherapy is a valuable therapeutic modality in the conservative treatment of BCC, and perilesional interferon (IFN) is included among these approaches. IFNs are a family of polypeptides with pleiotropic functions secreted by various cell types in the body. They are proteins belonging to a group of signaling molecules known as cytokines that participate in the regulation of the immune response [8, 9].

HeberFERON is a combination of IFNs alpha‐2b and gamma with antiproliferative, antiangiogenic, and immunomodulatory properties. The antitumor action of IFNs is mediate by inhibition of tumor cell growth and induction of apoptosis (programmed cell death). Both IFNs have antiangiogenic properties, which contributes to reducing the vascularity surrounding these tumors, and the combination of two active agents enhances the pharmacokinetics of the drug, as they may act synergistically [10, 11].

Among the benefits described for the use of IFNs is the possibility of conservative treatment that avoids reconstructive surgery and reduces the tumor or eliminates it completely [12].

This study was conducted with the aim of describing the results of HeberFERON administration in a series of patients with histologically aggressive BCC after 5 years of treatment.

## 2. Materials and Methods

### 2.1. Study Design and Setting

A descriptive, longitudinal, observational case‐series study was conducted in patients with a histopathological diagnosis of aggressive BCC who attended the dermatology clinic at the “Juana Naranjo León” University Polyclinic in primary healthcare. Participants were enrolled in the study during the period from September 2016 to October 2020. From the time of inclusion, each patient was followed for a period of at least five years.

### 2.2. Participants

The study group consisted of all patients with a histological diagnosis of aggressive BCC who attended dermatology consultations and in whom surgery was not performed for any of the following reasons: personal history of disease contraindicating the procedure, allergy to anesthetics, the tumor or its excision being likely to cause mutilation or deformity, or the patient’s refusal to undergo surgery. A total of 23 patients who received HeberFERON treatment were included.

The infiltrative, micronodular, morpheaform, basosquamous, and mixed histological subtypes were considered aggressive BCC. Tumor recurrence was defined as the reappearance of BCC in the same area where the primary tumor had been located, that is, at the site of a lesion previously treated with HeberFERON after a period of absence and at least 2 months after treatment.

### 2.3. Intervention

At the initial evaluation, a medical record was compiled including demographic and clinical data and physical examination findings. Initial photography, dermoscopy, and histopathological study by 3‐mm punch biopsy were performed to confirm the aggressive histological subtype; complementary tests were also indicated.

The therapeutic intervention was performed on an outpatient basis. The dose of HeberFERON was 10.5 × 10^6^ IU/mL, administered perilesionally and intradermally, 3 times per week for 3 weeks, until completing 9 doses.

### 2.4. Final Evaluation

This was performed at 16 weeks after the first dose to measure response to therapy. Clinical and dermoscopic parameters were taken into account. All information obtained was collected in the data collection notebook.

### 2.5. Follow‐Up

Follow‐up began from inclusion in the study. One month after completing treatment, the patient was assessed to evaluate laboratory results and lesion measurement. At 16 weeks after the start of therapy, the patient was evaluated and the treatment response was described. The duration of follow‐up for patients with histologically aggressive BCC was 60 months: every three months during the first year, every 6 months during the second year, and annually from the third to the fifth year. Each patient was informed that if a skin lesion was detected during the interval between one scheduled visit and the next, they should attend the clinic immediately and freely to be evaluated by the specialist.

### 2.6. Outcome Evaluation

This was performed at 16 weeks after the first dose and throughout the 5‐year follow‐up period.

### 2.7. Main Response Variables

The variables studied were clinical response to treatment at 16 weeks and recurrence at 5 years after treatment.

Clinical response to treatment in the case series was described according to the following criteria [13]:

• Complete response (CR): complete disappearance of the lesion.
• Partial response (PR): at least a 30% reduction in the sum of the largest diameters, taking as reference the baseline sum of the largest diameters.
• Stable disease (SD): reduction insufficient to qualify as PR or increase insufficient to qualify as progressive disease.

Dermoscopic response, using the DermLITE DL100 dermatoscope to evaluate dermoscopic structures characteristic of BCC and by comparing the initial and final images of the lesion, was classified as follows:

• Absence of tumor: no evidence of structures specific to BCC (pigmented, vascular, nonpigmented, and nonvascular structures and absence of pigment network) at the end of treatment.
• Presence of tumor: evidence of structures specific to BCC (pigmented, vascular, nonpigmented, and nonvascular structures and absence of pigment network) at the end of treatment.

Tumor recurrence was considered present when the tumor lesion reappeared in the same area where the BCC previously treated with HeberFERON had been located, after a period of absence and at least 2 months after treatment.

The time to occurrence of tumor recurrence was determined, and recurrence was treated according to the available therapeutic modalities and the patient’s characteristics.

### 2.8. Data Collection and Management

Information was obtained from the patient, biopsy reports, and results of complementary tests and recorded on a previously designed and prepared form. Follow‐up information for the patients was documented in this record. Once the primary data had been collected, a database was created to register them, after prior coding, in order to perform the corresponding statistical analyses.

### 2.9. Information Processing and Analysis Plan

Information was processed in an automated manner using SPSS Version 22.0 statistical software. Frequency tables were used to describe the sociodemographic, clinical, and histopathological characteristics of the patients. Mean values were used for age and tumor size. Treatment response according to the occurrence of recurrence was described by survival analysis based on the nonparametric Kaplan–Meier method. The mean time to occurrence of the outcome and its 95% confidence interval, as well as overall survival and its 95% confidence interval, were estimated.

### 2.10. Ethical Considerations

Patients and accompanying persons were informed of all procedures planned in the study and had the opportunity to discuss any questions about the research with the attending physician. Each patient signed an informed consent form. The data obtained were used for scientific purposes, and the identity of the included subjects was kept anonymous. This research adhered to the recommendations of the Declaration of Helsinki (1989), intended for those conducting research involving human beings and subsequently revised on several occasions. The project was approved by the Medical Ethics Committee and the Scientific Council of the Juana Naranjo León Center Polyclinic institution.

## 3. Results

Of the total patients included, 16 were male; skin phototype II predominated; the age range was between 51 and 85 years; the most common tumor location was the facial region, mainly the cheeks; the predominant aggressive histological subtype was infiltrative; and the predominant clinical subtype was ulcerative (Table 1).

**TABLE 1:** Baseline sociodemographic, histopathological, and clinical characteristics of the 23 patients with basal cell carcinoma of aggressive histological subtype.

Variable	No. (%)
Age^†^	71.87 ± 10.15 (51–85)
Sex	
Male	16 (69.5)
Female	7 (30.4)
Skin phototype	
Type I	1 (4.3)
Type II	13 (56.5)
Type III	8 (34.7)
Type IV	1 (4.3)
Lesion size^†^	23.86 ± 12.47 (0.8–60)
Clinical subtype	
Nodular	8 (34.7)
Ulcerated	14 (60.8)
Flat cicatricial	1 (4.3)
Aggressive histological subtype	
Infiltrative	11 (47.8)
Basosquamous	7 (30.4)
Micronodular	3 (13.0)
Morpheaform	2 (8.6)
Location	
Nose	3 (13.0)
Periocular	3 (13.0)
Cheeks	7 (30.4)
Temporal	4 (17.3)
Auricle	3 (13.0)
Upper limb	1 (4.3)
Anterior thorax	2 (8.6)
Previous treatment	6 (26.0)
Surgery	3 (13.0)
5‐Fluorouracil	2 (8.6)
Electrodessication and curettage	1 (4.3)
Multiple lesions (> 2)	3 (13.0)

^†^Mean ± SD (minimum–maximum).

**FIGURE 1:**
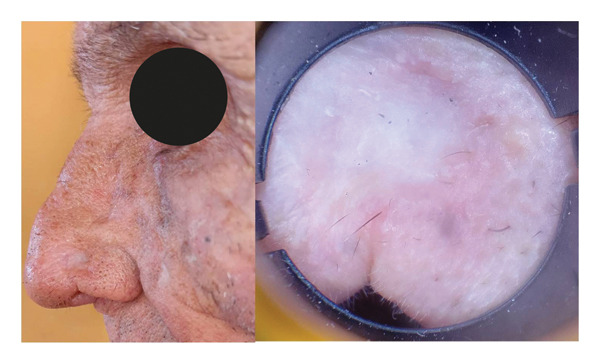
Clinical and dermatoscopic image of a patient with basosquamous carcinoma in the evaluation clinic.

**FIGURE 2:**
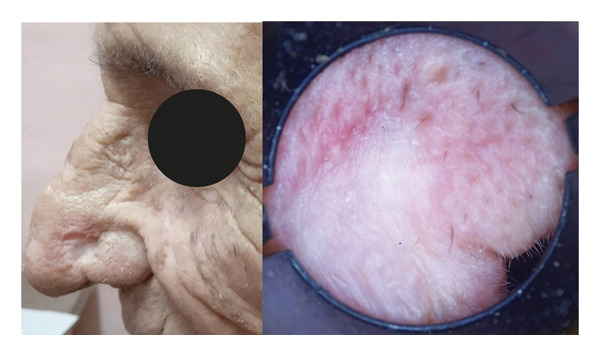
Clinical and dermatoscopic image of a patient with a basosquamous carcinoma 16 weeks after completing treatment with HeberFERON.

**FIGURE 3:**
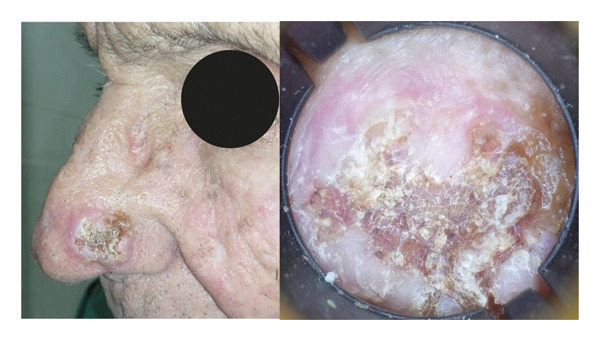
Clinical and dermatoscopic image 5 years after treatment with HeberFERON.

Table 2 shows the clinical response to HeberFERON treatment at 16 weeks. CR was observed in 14 patients, corresponding to 63.6% (95% CI, 39.2–82.4), with an objective response in 90.9% and disease control in 100% of the cases (Figures 1, 2, and 3).

**TABLE 2:** Clinical response at 16 weeks in 22 patients with basal cell carcinoma of aggressive histological subtype treated with HeberFERON.

Response to treatment	Response at 16 weeks to treatment with HeberFERON	N (%)	95% CI
Clinical response	Complete response (CR)	14 (63.6)	39.2–82.4
	Partial response (PR)	6 (27.3)	
	Objective response (RC + RP)	20 (90.9)	
	Stable disease (SD)	2 (9.1)	
	Disease control (RC + RP + EE)	22 (100)	

**FIGURE 4:**
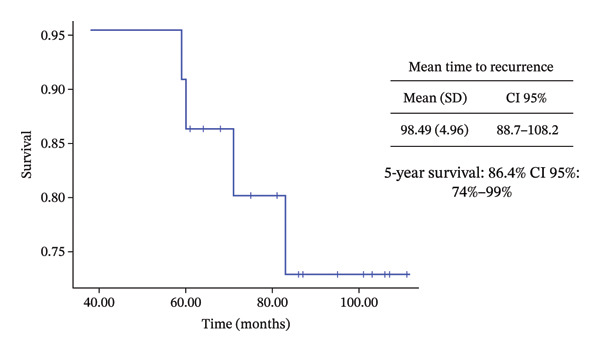
Survival analysis for tumor recurrence in 22 patients with histologically aggressive basal cell carcinoma treated with HeberFERON.

Figure 4 shows the survival analysis for tumor recurrence in the 22 patients. The mean time elapsed until the appearance of recurrence was 98 months (95% CI, 88.7–108.2). The probability of remaining recurrence free at 5 years was 86.4% (95% CI, 74%–99%).

## 4. Discussion

The incidence of BCC has increased in recent years and constitutes a health problem with social impact, given the psychological, esthetic, and functional implications it may cause [14].

From 2016 to 2020, 23 patients with aggressive‐histology BCC were evaluated. The epidemiological data of the patients are similar to those reviewed in the literature. Incidence doubles in men older than 40 years, and involvement predominates in patients with skin phototype II. The facial region was the location with the highest number of BCCs in the series; other authors obtained similar results [15, 16].

In this study, the infiltrative aggressive histological subtype and the ulcerative clinical subtype predominated (ulceration may indicate the aggressive nature of the tumor). This phenomenon was also identified by some authors with similar results; however, for others, the morpheaform histological subtype was more common [17–19].

Several international articles report the effectiveness of IFNs in BCC, and around a dozen Cuban articles confirm the efficacy of HeberFERON in this tumor. IFNs suppress cell proliferation, increase macrophage phagocytosis, inhibit angiogenesis, and enhance the cellular immune response of T lymphocytes [20–26].

Clinical response rates to perilesional HeberFERON in Cuba for BCC range from 71% to 100%. CR in this study was 63.6% with a 95% CI of 39.2–82.4; this result falls within the figures cited in the articles. PR was 27.3%, so this outcome is within the range found in the consulted literature (11%–35%) [24–26]. The articles previously referenced combine BCCs with different histologies, whereas this series includes only those with aggressive histology.

To consider a patient cured, follow‐up for 5 years or more is necessary to detect tumor recurrence; this variable is considered the most relevant and recommended primary outcome in systematic reviews and meta‐analyses [27]. Most published articles on BCC and IFN report follow‐up only up to 16 weeks [12, 28, 29]. All subjects in this study were followed for 5 years; two patients (9.0%) discontinued the follow‐up. Recurrence was evaluated by Kaplan–Meier survival analysis. Survival analysis showed that the mean time to recurrence was 98 months (95% CI, 88.7–108.2). At five years, 86.4% (95% CI, 74%–99%) of the subjects had the probability of remaining recurrence‐free.

In a multicenter study in patients with high‐risk facial BCC, 13 subjects had aggressive histology with predominance of the infiltrative variant (similar to our study), and CR was 73.3%. Survival analysis showed that 12.3% developed tumor recurrence with a mean time of 55 months [30]. Similar results were obtained in the present study, with the difference that the time to recurrence was longer (98 months).

A study conducted in Gothenburg, Sweden, included 15 patients with an age range similar to the present series although women predominated, with aggressive BCCs (primary or recurrent morpheaform and infiltrative tumors), all located on the face, treated with intralesional IFN alpha‐2b and followed for 6–30 months. This interferon treatment was administered before Mohs micrographic surgery (MMS). In 27% of the patients, no tumor was detected when MMS was performed; in 33%, the tumor was reduced by more than 75%; and the remaining six patients (40%) did not respond to IFN treatment. The authors reported one recurrence 1 year after treatment and a mean follow‐up of 18 months [31]. Follow‐up in the present series was 5 years, cure at the end of treatment was higher, and although the previously mentioned study refers to one patient with recurrence, the number of treated cases was smaller and follow‐up lasted only one and a half years.

Bostanci and collaborators reported seven‐year follow‐up of 13 patients with BCC, 10 with aggressive histology (seven micronodular and three infiltrative) treated with IFN alpha‐2b. Fifty‐five percent of the lesions showed CR, 30% PR, 10% of the tumors showed no response to treatment, and one lesion increased during therapy. At 5 years, one infiltrative micronodular BCC presented tumor recurrence [32]. The results of that study were inferior to those presented in this series, which may perhaps be explained by the fact that HeberFERON is a combination of alpha‐2b and gamma IFNs because it is composed of two IFNs. It produces increased and prolonged pharmacological activity without additional toxicity, with a faster and more prolonged effect compared with individual IFNs [33, 34].

A study carried out in patients with nasally located tumors and aggressive histological subtype (80% infiltrative) who received perilesional IFN alpha‐2b confirms cure in all cases with good esthetic results. After 4 years of follow‐up, the authors reported no tumor recurrence and adverse events were mild to moderate, concluding that this drug may be a therapeutic alternative for this anatomic region [35].

The results of our study were similar although unlike the article mentioned above, the follow‐up was 5 years, and not only BCCs with infiltrative histology were included but also micronodular, morpheaform, and basosquamous subtypes were incorporated into the sample, the latter with a high tendency toward recurrence and metastasis, implying an unfavorable prognosis [36].

This is the first reported study to include only BCCs with all aggressive histological subtypes treated with HeberFERON in primary healthcare with 5 years of follow‐up. The evidence obtained may be useful for decision‐making regarding this type of tumor in the global population. As this was a descriptive study, it did not allow comparisons with other therapeutic modalities.

## 5. Conclusions

HeberFERON may be considered a first‐line treatment for BCC with aggressive histology and as a therapy prior to surgery. Cure was achieved in most patients, and 5‐year follow‐up allowed recurrence to be detected in some cases.

## Author Contributions

The study concept and design: Vladimir Sánchez Linares and Vicente Eloy Fardales Macías.

Data collection, analysis, and interpretation: Vladimir Sánchez Linares and Iraldo Bello Rivero.

Statistical analysis: Iraldo Bello Rivero, Vladimir Sánchez Linares, and Vicente Eloy Fardales Macías.

Writing of the manuscript: Vladimir Sánchez Linares and Vicente Eloy Fardales Macías.

Effective participation in the research guidance: Vladimir Sánchez Linares and Iraldo Bello Rivero.

Intellectual participation in the therapeutic conduct of the studied cases: Vladimir Sánchez Linares.

Critical review of the literature: Vladimir Sánchez Linares and Iraldo Bello Rivero.

Final approval of the version of the manuscript: Vladimir Sánchez Linares, Iraldo Bello‐Rivero, and Vicente Eloy Fardales Macías.

## Funding

This research did not receive any specific grant from funding agencies in the public, commercial, or not‐for‐profit sectors.

## Ethics Statement

The Institutional Ethical Board of Polyclinic Juana Naranjo Leon approved the protocol for this study. All participants gave their written informed consent. The procedures and data management were in accordance with Good Clinical Practice guidelines and the ethical principles stated in the Declaration of Helsinki.

The patients in this manuscript have given written informed consent to publication of their case details.

## Conflicts of Interest

The authors declare no conflicts of interest.

## Data Availability

The data that support the findings of this study are available from the corresponding author upon reasonable request.
